# Adopting Bacteria in Order to Adapt to Water—How Reed Beetles Colonized the Wetlands (Coleoptera, Chrysomelidae, Donaciinae)

**DOI:** 10.3390/insects2040540

**Published:** 2011-12-09

**Authors:** Birgit Kleinschmidt, Gregor Kölsch

**Affiliations:** Zoological Institute, Molecular Evolutionary Biology, University of Hamburg, Martin-Luther-King-Platz 3, 20146 Hamburg, Germany; E-Mail: birgit.kleinschmidt@gmx.net

**Keywords:** Donaciinae, symbiosis, gamma-proteobacteria, co-speciation, tetracycline, pupation rate, cocoon formation

## Abstract

The present paper reviews the biology of reed beetles (Donaciinae), presents experimental data on the role of specific symbiotic bacteria, and describes a molecular method for the detection of those bacteria. Reed beetles are herbivores living on wetland plants, each species being mono- or oligo-phagous. They lay their eggs on the host plant and the larvae live underwater in the sediment attached to its roots. The larvae pupate there in a water-tight cocoon, which they build using a secretion that is produced by symbiotic bacteria. The bacteria are located in four blind sacs at the foregut of the larvae; in (female) adults they colonize two out of the six Malpighian tubules. Tetracycline treatment of larvae reduced their pupation rate, although the bacteria could not be fully eliminated. When the small amount of bacterial mass attached to eggs was experimentally removed before hatching, symbiont free larvae resulted, showing the external transmission of the bacteria to the offspring. Specific primers were designed to detect the bacteria, and to confirm their absence in manipulated larvae. The pupation underwater enabled the reed beetles to permanently colonize the wetlands and to diversify in this habitat underexploited by herbivorous insects (adaptive radiation).

## 1. Introduction

Symbioses are common in the living world [1-5]. In addition to macroscopically evident symbioses (like of crabs and anthozoans, [6]), ongoing research reveals more and more examples of predominantly mutualistic relationships, in which plants and animals live together with microorganisms. Bacteria, fungi and other eukaryotic protists can be involved as symbionts [7,8], which live either in close association with or within their respective host. In the latter case, they can live intra- or extra-cellularly. Even if we confine this initial overview to insects and their bacterial endosymbionts, we still find a great number of systems [9-11], although much of the research has so far concentrated on few classical examples. As an effect of the symbiosis, the bacteria can expect a stable environment, and physiological studies showed that they obtain various metabolites from the host cell. The benefit to the host is usually assumed to be nutritional. Typically, such symbioses can be found in insects that live on a restricted diet deficient in one or more type(s) of nutrients [12-15]. Hence, the evolutionary innovation achieved is usually the exploitation of a new food source. Although concentration on these systems may have caused a bias in the spectrum of interactions, the low number of interactions other than mutual exchange of nutrients seems to be real. Benefits beyond food and shelter include protection from pathogens and increased temperature resistance [16-23].

In the system we describe here, the innovation is the exploitation of a new habitat, namely the wetlands. Reed beetles (Chrysomelidae, Donaciinae) were able to colonize wetlands permanently, because the larvae pupate in a cocoon underwater, which they build using a secretion produced by their symbiotic bacteria [24]. We review the known details of this association and discuss the consequences for reed beetle evolution. The experimental part provides new data on the role the bacteria play, adding to the hitherto indirect evidence (see below). Given that the bacteria are involved in the production of a secretion required for pupation, we tested the hypothesis that larvae lacking those bacteria would be unable to pupate. We established and describe a molecular method for the detection of the symbionts applicable also to early instar larvae, which is at the same time a proof of the transmission pathway.

### 1.1. Reed Beetle Biology

**Figure 1:**
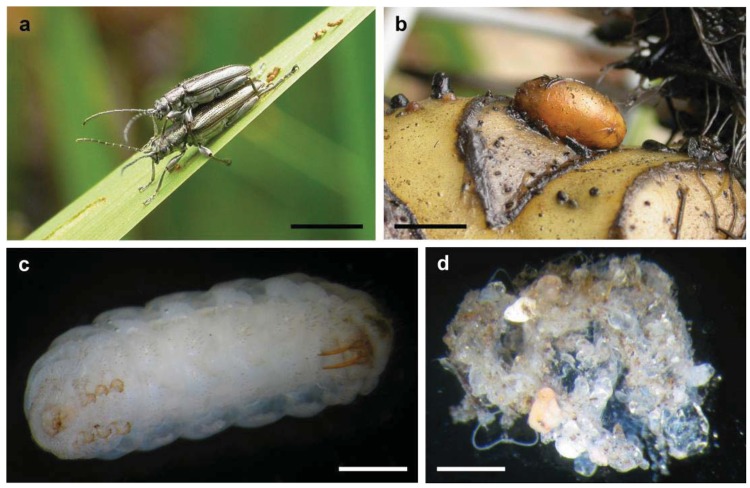
(**a**) Adults of *Donacia* cf. *subtilis* in pre-mating position; (**b**) Cocoon of *Donacia* sp. (probably *D. cincticornis*) on a water lily rhizome; (**c**) Late instar larva of *Macroplea mutica*, ventral view; The small head, three pairs of rudimentary legs, and the pair of abdominal stilettos (on the right, see text) are visible (**d**). Phase 1 cocoon material with adhering sand, preserved in ethanol; the thin membranous material is visible between sand grains in the centre and lower right part of the formation. Scale bars: 5 mm in (**a**) and (**b**), 1 mm in (**c**) and (**d**).

Reed beetles (Donaciinae) are a relatively basal group within the leaf beetles (Chrysomelidae) [25-27]. Their habitus is similar to that of long-horned beetles (Cerambycidae): the antennae are long, and the “shoulders” (humeral angles of the elytra) are prominent (Figure 1a). Morphologically, they are well defined by the first abdominal sternite being as long as all following ones together [28]. The approximately 165 species show a Holarctic distribution, with some species occurring in Africa, Central America and Northern Australia [29-31]. Reed beetles live on herbaceous plants in wetlands, from wet sedge meadows to submerged vegetation, with most species being mono- or oligo-phagous. Adult beetles feed on the leaves of their host plant although some species are pollen feeders. The females lay the eggs in the lower parts of the plants, for example between leaf and stem, often underwater. The larvae (Figure 1c) live attached to the roots in the sediment [32,33]. Their mode of feeding is discussed in detail by Böving [32], and most if not all species live as sap suckers gnawing a hole into the root. They breathe by tapping the aerenchyme of the plant with two hollow abdominal stilettos, which are connected to their tracheal system [32,34]. The larvae pupate at the end of their second summer in a cocoon (Figure 1b). The beetle overwinters in the air filled cocoon and ecloses in the following spring. In warmer climate the larvae may pupate after their first summer. Information on the formation of the cocoon is summarized by Böving [32]. The epidermis of a mature larva produces a waxy secretion that covers the entire body prior to pupation. The larva lines this preliminary construction on the inner side using a secretion oozing from its mouth while performing spinning movements. It is not clear to which extent excretions from the anus are further involved. This explains why the wall consists of several thin layers. The completed cocoon only consists of layers produced during this second phase (14–18 in *Donacia brevicornis*, each 0.3 to 1.4 μm thick; [32,35]). The secretion used during the first phase is ephemeral. In late summer, cocoons isolated from sandy sediment sometimes have sand grains adhering to them, which can easily be wiped off (Figure 1d, personal observation G.K.). Older cocoons are devoid of such covering, separating from sediment in a perfectly clean manner (Figure 1b). The cocoon material is flexible and resists strong alkaline and acid solutions [32], although warm KOH leads to a partial degradation [35]. Fibrils present in the cocoon wall are predominantly oriented at 90° and 45° to the long axis of the cocoon [36]. Cocoons from at least the preceding year can be found in the field. While the waxy secretion produced during the first phase could be similar (homologous) to material used by other chrysomelid larvae for stabilizing their pupal chambers in the sediment, the phase two secretion is as unusual as the formation of a cocoon *per se* among chrysomelid beetles. The composition of the cocoon material is not precisely known, but preliminary results point at quinone tanned protein [35].

### 1.2. The Association with Bacteria Providing Cocoon Material

According to all evidence available so far, the cocoon material is produced by endosymbiotic bacteria of the larvae. The larvae possess four blind sacs at the foregut, which become more and more prominent as the larvae mature. Those organs were first described by Hirschler [37], who interpreted these as equivalent to hepatopancreatic glands of the Crustacea. Stammer [38] re-evaluated the structures and realized that they were in fact bacteriomes. His histological images show the bacteriocytes filled with intracellular bacteria. He investigated the entire synchronized life cycle of host and bacteria, during which the bacteria colonize specific parts of Malpighian tubules of the old larva. He also observed that the blind sacs are reduced in the pupa. The symbionts belong to the Enterobacteriaceae within the gamma-proteobacteria, more specifically to a clade containing many symbionts of insects and other metazoans [39]. The evidence hitherto available that the bacteria are involved in the production of the secretion can be summarized as follows [39]: There are large amounts of a viscous fluid in the blind sacs (see also [37,40]) and in the digestive tract of larvae ready to pupate. This secretion is used by the larvae for building the cocoon. The host cells in the blind sacs do not show any differentiation into gland cells and are not capable of producing the fluid.

### 1.3. Transmission Pathway of the Bacteria

If the bacteria are so crucial for the development of the beetles, one should expect a mechanism by which reliable transmission is ensured. Such a mechanism can be found in the Donaciinae. The female deposits a small droplet of bacterial mass on one end of the egg during oviposition. The hatching larva inevitably ingests the bacteria and thereby infects itself [38]. Larvae of both sexes harbor the symbionts. In the adult beetle, they only occur in two out of the six Malpighian tubules. Here, only certain parts of the tubule are swollen, because the cells contain bacteria in great number [38,39]. In the males of some species, this swelling of the tubules is very small or even absent [38] (personal observation G.K.). This supports the concept that only the larvae require the symbionts, and the females have to accomplish the transmission, while in males a complete loss is possible.

## 2. Experimental Section

To obtain symbiont-free larvae to test for effects on pupation, we treated the animals with the antibiotic tetracycline. This substance is routinely used in the treatment of bacterial infections in aquatic animals and in similar studies on insects [41] and thus appeared suitable in the case of the aquatic reed beetle larvae. Tetracycline inhibits the protein biosynthesis at the level of the translation [42]. Larvae of *Macroplea* sp. were collected in summer in a lake where *Macroplea mutica* (Fabricius, 1792) and *M. appendiculata* (Panzer, 1794) occur syntopically. Those larvae had hatched during the preceding summer and could be expected to pupate during the experimental summer season. The larvae were allocated to three size classes according to their body weight (size class 1: <15 mg; 2: 15–25 mg; 3: >25 mg). They were kept in aquaria filled 10 cm high with sand and equipped with host plants (*Potamogeton perfoliatus* and *P. pectinatus*) at ambient temperature and light conditions. For each size class, two aquaria were set up (treatment and control; for sample sizes see Table 1). After three days of acclimation, tetracycline was applied in the treatment groups over one week directly in the aquarium water: 8 mg/L on day 1, 6.5 mg/L on day 2, and 5.5 mg/L on each of five consecutive days. After the treatment, half of the water was substituted by a fresh mixture of lake and tap water. In autumn (after 63 days, on 21 September 2011), the sediment and the plants were searched for larvae and cocoons, which were collected and stored at −20 °C. The pupation data were analyzed using the G-test, in the case of 2 × 2 tables with William's correction (yielding an adjusted G-value we call Gadj [43]).

We also used a second method for obtaining aposymbiotic larvae. This involved mechanical removal of the bacterial mass adhering to the eggs, in order to prevent the self-infection of larvae during hatching. To obtain the eggs, *Donacia marginata* (Hoppe, 1795) and *Donacia semicuprea* (Panzer, 1796) were kept in small groups of pairs in cages containing their respective host plant in water (*Sparganium ramosum* and *Glyceria maxima*), on which the females laid eggs in small clutches underwater or close to the water line on wet leaf material. Every two or three days, the eggs were carefully removed and kept floating on water in Petri dishes, the bottom of which was covered with 1% agar. The bacterial mass adhering to the anterior pole of the egg was removed: The eggs are embedded in a rubber-like mass during oviposition, which quantitatively contains the bacteria [44] and from which the egg can be isolated without any material remaining on the smooth surface of the egg. Unmanipulated eggs were kept as a control. The Petri dishes were checked daily for hatched larvae, which were then collected and frozen.

**Figure 2:**
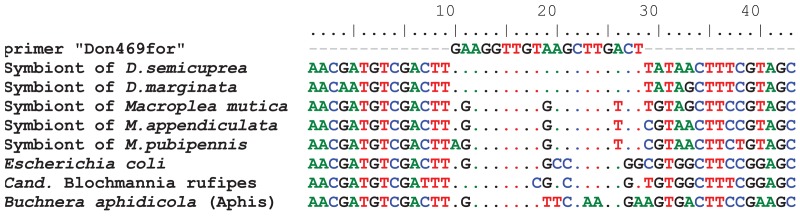
Alignment of the primer Don469for to the corresponding partial sequence of the 16SrRNA of symbionts of *Donacia* and *Macroplea* species, as well as sequences of three further Enterobacteriaceae, which show different levels of relatedness to reed beetle symbionts (cf. [39]).

In order to obtain the organs containing the symbionts, larvae were dissected in 100% ethanol. The foregut blind sacs and Malpighian tubules were removed and kept separately. The small newly hatched larvae (*ca.* 2 mm) were not dissected, but used *in toto*. At least five of them had to be pooled in order to obtain enough DNA for reliable diagnoses. For DNA extraction, tissues/larvae were frozen in liquid nitrogen, ground in a reaction tube, and suspended in 180 μL enzymatic lysis puffer containing lysozyme, as described in the handbook of the DNeasy blood & tissue kit (Qiagen, Hilden). Following incubation at 37 °C for 1.5 hours, the samples were treated according to the instructions for animal tissues in that kit, with an elution volume of twice 25 μL. For the detection of the symbionts via diagnostic PCR, specific primers were developed. For the symbionts of the beetle genus *Macroplea*, the oligonucleotide used for fluorescent *in situ* hybridization was adopted (Mac941rev; 5′-GAGGATGCTGCCCTTTGTA-3′; [39]). It targets the 16S rRNA and was used in conjunction with the universal bacterial primer Tbac357for (5′-CTCCTACGGGAGGCAGCAG-3′; [39]), yielding a product of *ca*. 900 base pairs in length. For symbionts of *Donacia*, a different 16S region was used, where the symbionts of the two beetle species used were identical, but differed in at least three positions from *Macroplea* symbionts and other related bacteria (Figure 2). This primer Don469for (5′-GAAGGTTGTAAGCTTGACT-3′) was used together with bac1492rev (5′-TACGGYTACCTTGTTACGACTT-3′; [39]), resulting in a *ca.* 600 bp fragment.

The touchdown PCR protocol, optimized in a gradient PCR, for the primers Don469for and Tbac1492rev was as follows: initial denaturation at 95 °C for 5 min, 17 cycles with 95 °C for 30 s, annealing for 1 min at 60 °C (decreasing with each cycle by 0.5 °C), elongation at 72 °C for 80 s. This was followed by 24 cycles with 51 °C constant annealing temperature. A final elongation lasted 5 min. For the primers Tbac357for and Mac941rev a simple PCR protocol was used (92 °C for 1 min, 68 °C for 1 min and 72 °C for 1 min, 34 cycles). Each PCR reaction contained 2 μL extracted DNA, 2.5 μL 50 mM MgCl_2_, 5 μL 10× buffer, 4 μL of each primer (10 μM), 1 μL dNTPs (10 mM each) and 0.25 μL Taq DNA polymerase (invitrogen, 5 U/μL). In the case of the newly hatched larvae, 3 μL DNA extract had to be used for reliable detection of symbionts. The volume was adjusted to 50 μL using ultra-pure water. Negative and positive control reactions were run together with the samples. PCR products were visualized in a 1% agarose gel containing ethidium bromide under UV light. To verify the specificity, randomly selected PCR products were purified (QIAquick PCR purification kit, elution with 50 μL) and sequenced on an ABI sequenator 3100 using the BigDye technology. The sequences were edited using Sequencher (Gene Codes) and used for BLAST searches in GenBank.

## 3. Results and Discussion

### 3.1. Antibiotic Treatment Reduces the Pupation Rate

Direct evidence for the central role the bacteria play for cocoon formation comes from the experiment with tetracycline treated larvae. Of the 75 larvae used in the tetracycline experiment, 64 were recovered at the end. Survival did not differ between treated group (30 out of 35) and control (34 out of 40) (Gadj = 0.01, 1 df, n.s.). We found free larvae, cocoons with larvae (=filled) and empty cocoons (Table 1). The pupation frequency was significantly reduced in the treatment group (G-test: No. of free larvae (minus No. of empty cocoons) *versus* filled cocoons, in treatment and control group; Gadj = 4.21, 1 df, p ≤ 0.05). However, there was no all-or-nothing response, as some larvae pupated in spite of the tetracycline treatment. This had also been the case in the preliminary work mentioned by Kölsch and Pedersen [45]. The empty cocoons deserve attention. There were more empty cocoons than filled ones in the treatment group (8 *versus* 3) and the other way round in the control group (4 *versus* 19). These two patterns are significantly different (Gadj = 9.45, 1 df, p ≤ 0.01). It might be that the treated larvae abandoned a cocoon they could not complete due to a lack of building material.

**Figure 3:**
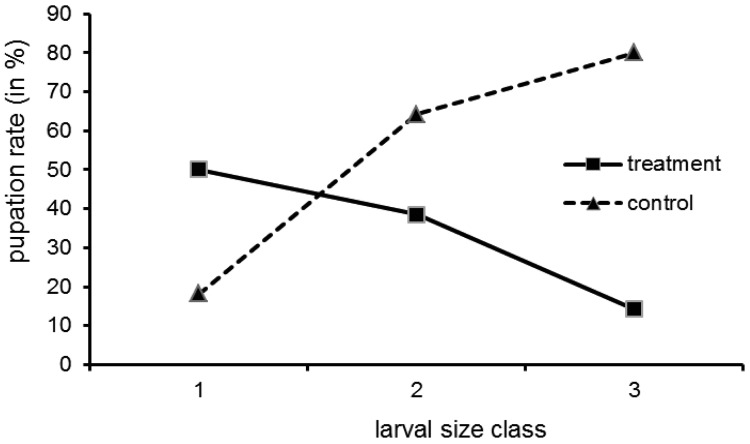
Pupation rate of *Macroplea* larvae of different size at the beginning of the experiment, after tetracycline treatment and without application of antibiotics (control), respectively. Size classes are defined in the text, sample sizes are given in Table 1.

Pupation was not uniformly distributed over the larval size classes (Figure 3; all size data refer to the situation at the onset of the experiment). While in the control group the pupation rate increased with size class, in the treatment group the opposite was the case. The interpretation is a follows: the small larvae may not have reached maturity by the time the experiment was finished (which explains a moderate to low pupation rate in both groups). Still, for small larvae in the treatment group the pupation rate is higher than for bigger ones, because in the larvae treated at an earlier stage the bacteria could recover. The bigger larvae could not pupate, because when they reached maturity relatively shortly after treatment, the bacteria had not proliferated in the organs relevant for pupation (blind sacs) due to the antibiotic. Tetracycline, although successfully used in similar studies, is only a cytostatic, and over the two months of our experiments the bacteria may have regained their full metabolic activity in the small larvae.

### 3.2. Cured or not—the Molecular Diagnosis

**Figure 4:**
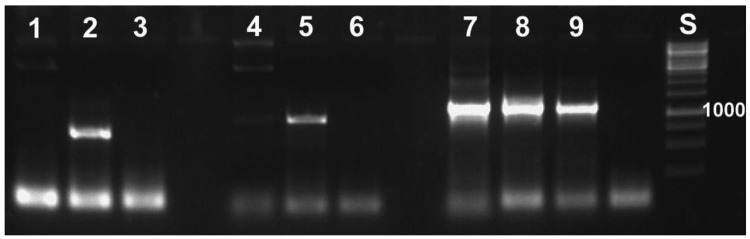
Specific primers developed for reed beetle symbionts do not yield a PCR product with standard laboratory strains of *E. coli* (1% agarose gel containing ethidium bromide, UV illumination). Primers used: 1–3: specific for *Donacia* symbionts; 4–6: specific for *Macroplea* symbionts; 7–9: general bacterial primers. The samples are: 1, 4, 7: *E. coli*; 2, 8: *D. semicuprea* symbiont; 3: negative control (distilled water); 5, 9: *Macroplea* sp. symbiont; 6: negative control; S: size standard with the fragment size 1,000 bp given (cf. Figure 6).

With the molecular tools developed as described above, we were able to specifically detect the symbionts. This specificity was required, because it is not possible to work under sterile conditions that would avoid contamination by, for example, bacteria contained in the digestive tract of the animal. Application of the primers to samples of *Escherichia coli* did not yield PCR products (Figure 4). All PCR-products sequenced revealed reed beetle symbionts in the BLAST searches conducted. All best hits were sequences of *Candidatus* Macropleicola, the symbionts of the reed beetle genus *Macroplea* (accession no. GQ480915 and GQ480918 [39]), and the bacterial symbionts of *Donacia semicuprea* (accession no. GQ480891, GQ480897, GQ480898, GQ480907, GQ480936; [45]), respectively, consistent with the host species used.

**Figure 5:**
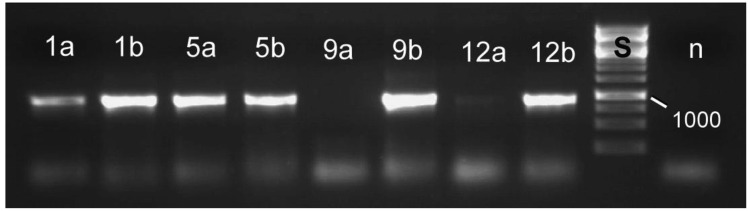
Molecular detection of symbiotic bacteria in larvae of *Macroplea* sp. from the tetracycline experiment, using specific PCR primers. Larvae of all four groups (tetracycline treated/control × free larvae/larvae from cocoon) contained symbiont DNA. Samples are: 1: free larva, tetracycline treated; 5: free larva, not treated; 9: larva from cocoon, tetracycline treated; 12: larva from cocoon, not treated; a: DNA extracted from blind sacs; b: DNA extracted from Malpighian tubules. The absence of symbionts from the blinds sacs of larvae after cocoon formation (9a, very faint band in 12a) was not always found, but is in compliance with the pattern of host tissue colonization [41]. n: negative control, distilled water as sample; S: size standard with the fragment size 1,000 bp given (cf. Figure 6).

To test the larvae from the pupation experiment for symbiotic bacteria, DNA from both treated and control larvae were extracted. Both free larvae and those from cocoons were used. We were able to detect symbionts in all four groups, although sometimes only one of the organs (blind sacs/Malpighian tubules) was positive. In particular, presence of bacteria in tetracycline treated larvae was confirmed (Figure 5). We cannot judge if the bacteria were metabolically active, or if simply the DNA of inactive bacteria was detected. However, the results explain the high incidence of pupation due to incomplete elimination of the bacteria. These results show that a more effective way of eliminating the bacteria is required (see Section 3.3).

### 3.3. Molecular Proof of Concept: External Transmission

**Figure 6:**
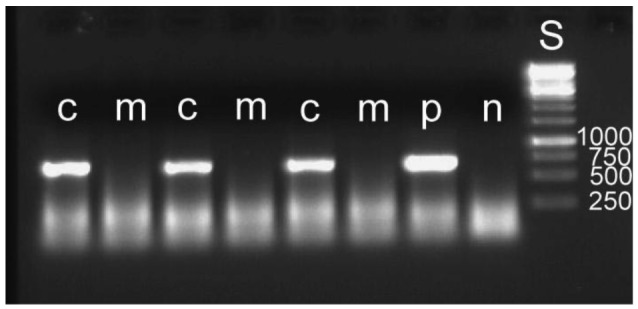
Molecular detection of symbiotic bacteria in larvae of *Donacia* sp. using specific PCR primers. In the manipulated samples, the bacterial mass adhering to the egg had been removed before hatching. c: control samples from normally hatched larvae; p: positive control, sample known to contain symbiont DNA; m: manipulated sample; n: negative control, distilled water as sample; S: size standard with the fragment sizes given.

The removal of the bacterial mass attached to an egg leads to a reduced overall hatching rate. Out of 510 manipulated eggs, 217 hatched, whereas 333 of 508 intact eggs hatched (Gadj = 54.64, 1 df, p ≤ 0.001). It cannot be determined what caused this higher mortality. It may be due to minute injuries of the membranous egg shell. For the detection of symbiont DNA in the larvae, five to seven larvae were pooled for DNA extraction. Eleven such groups were used for both manipulated and control larvae. In none of the manipulated groups could bacteria be detected, whereas this was possible in all control groups (examples: Figure 6). The results provide experimental evidence for the mode of external transmission of the bacteria as described by Stammer [38] (see above). There is no internal transmission of symbionts, which is found in some systems [1,46,47]. Another example with a strictly external transmission is the stinkbug *Megacopta punctatissima* [48].

### 3.4. Co-Cladogenesis of Beetles and Bacteria

**Figure 7:**
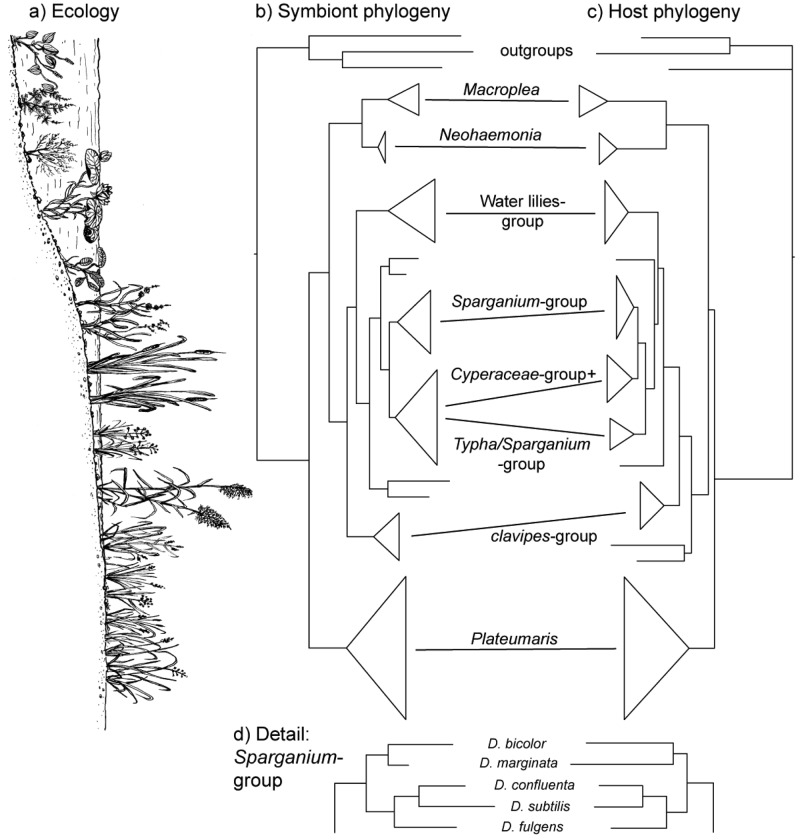
Co-cladogenesis of symbiotic bacteria and their reed beetle hosts. (**a**) Profile of the margin of a water body with characteristic vegetation; (**b**) Phylogenetic tree of the symbionts; (**c**) Phylogenetic tree of the hosts (reed beetles); (**d**) Congruent branching pattern within the *Sparganium*-group. The trees in (**b**) and (**c**) are schematic representations of the phylogenies presented in greater detail in [45]. The size of the triangles (length of vertical edge) is proportional to the number of species that had been included in that analysis (for scale: five species in the *Sparganium*-group). The species groups (names given between (**b**) and (**c**) and the position of four single species varying between methods used for tree construction are discussed in [55]. The position of the groups relative to the shore profile in (**a**) reflects their approximate habitat preference.

The vertical transmission from female to offspring makes horizontal transfer of bacteria between species or via the environment highly unlikely, if not impossible. Since this association has existed since the origin of the reed beetles (see below), hosts and symbionts share 75–100 million years of common history. The consequence is a pattern of strict co-cladogenesis (Figure 7). The congruence of the phylogenetic trees is not simply a superficial resemblance, but it extends to the very twigs of the trees, as exemplified by the species group occurring on bur-reed (*Sparganium*; Figure 7d). Co-cladogenesis is characteristic of the ancient accociations between insects and their primary endosymbionts [49,50]. We do not use the term co-evolution in this context [51,52], because it is not possible to provide evidence for a pattern of adaptations and counter adaptations that would be characteristic of co-evolution in the strict sense [53]. The term co-speciation, as used by Kölsch and Pedersen [45], implies—as pointed out by the authors—that one assumes species status not only for the beetle taxa, but also for the respective bacterial symbionts. This approach appears justified given the consistent genetic differences (16S sequences; [45]) and was followed in the formal description of two symbiont species from two *Macroplea* species [39]. Further characterization of the symbionts for example by multi-locus sequence typing/analysis (MLST; [54]) would shed more light on this aspect.

### 3.5. Evolutionary Consequences—Adaptive Radiation

Much of insect biodiversity is terrestrial. However, some insect orders have aquatic larvae (Ephemeroptera, Odonata, Plecoptera, Megaloptera, Trichoptera and certain Diptera), in others we find specialized groups with aquatic larvae and adults (Heteroptera, Coleoptera). The total number of insect species associated with water at least at some stage is about 60,000 (compiled after [33,56,57]), compared to more than 1 million described insect species in total. The permanent colonization of wetlands by insects is hampered by the necessity to return to land at some stage of their life cycle. The reed beetles could overcome this requirement. The larvae can live even in anoxic mud, because they use the oxygen contained in the root aerenchyme (see above). The second milestone is the cocoon in which they pupate. It is a self-made microenvironment that protects from water, mud and mechanical damage. It is air filled and thereby renders both metamorphosis and hibernation of the beetle in an aquatic environment possible.

During the early evolution of reed beetles (Cretaceous and early Tertiary), wetlands presumably were much more abundant than currently [58]. When the Donaciinae first appeared 75–100 million years ago [55], the relevant food plants in wetlands already existed [59,60]. That means that the beetles could colonize the entire range of habitats and plants available within the wetlands. The formation of a cocoon therefore was the key innovation [61] that led to an adaptive radiation. This radiation is mirrored by the early specialization on certain host plants, which formed the basis of the modern species groups within the reed beetles, which can be characterized by their host plant use [55].

## 4. Conclusions

The example of the reed beetles shows how a symbiosis between intracellular bacteria and an insect host can provide the basis for an innovation that eventually promoted the diversification of reed beetles during their colonization of wetlands, a habitat hitherto underexploited by insects. What remains obscure is the origin of this symbiosis. It is possible that a similar association was or is present in other beetles, which then developed from the preliminary stage into this final association. The detection of a similar constellation (similar bacterial symbionts and cocoon formation) in another group of beetles would help us to understand the path of reed beetle evolution.

## Figures and Tables

**Table 1:** Experimental setup and results of the tetracycline treatment of *Macroplea* larvae.

**Size Class**	**Start of Experiment**		**End of Experiment: No. of**		
	**Treatment**	**No. of Larvae**	**Free Larvae**	**Empty Cocoons**	**Cocoons with Larva**
1	+ tetracycline	8	8	4	0
	control	11	9	0	2
2	+ tetracycline	13	11	4	1
	control	14	4	2	7
3	+ tetracycline	14	8	0	2
	control	15	2	2	10

total	+ tetracycline	35	27	8	3
	control	40	16	4	19
